# *GhCLCc-1*, a Chloride Channel Gene from Upland Cotton, Positively Regulates Salt Tolerance by Modulating the Accumulation of Chloride Ions

**DOI:** 10.3390/genes15050555

**Published:** 2024-04-26

**Authors:** Wenhao Li, Siqi Gao, Yinghao Zhao, Yuchen Wu, Xiaona Li, Jianing Li, Wei Zhu, Zongbin Ma, Wei Liu

**Affiliations:** Collaborative Innovation Center of Henan Grain Crops, College of Agronomy, Henan Agricultural University, Zhengzhou 450002, China; liwh19970524@163.com (W.L.); 15037293611@163.com (S.G.); zhaoyinghao0521@163.com (Y.Z.); 17629992261@163.com (Y.W.); 13835278721@163.com (X.L.); 15716302681@163.com (J.L.); wei.zhu@henau.edu.cn (W.Z.); zongbinma@henau.edu.cn (Z.M.)

**Keywords:** *Gossypium hirsutum* L., salt stress, ionic toxicity, subcellular localization, functional analysis

## Abstract

The ionic toxicity induced by salinization has adverse effects on the growth and development of crops. However, researches on ionic toxicity and salt tolerance in plants have focused primarily on cations such as sodium ions (Na^+^), with very limited studies on chloride ions (Cl^−^). Here, we cloned the homologous genes of *Arabidopsis thaliana AtCLCc*, *GhCLCc-1A/D*, from upland cotton (*Gossypium hirsutum*), which were significantly induced by NaCl or KCl treatments. Subcellular localization showed that GhCLCc-1A/D were both localized to the tonoplast. Complementation of *Arabidopsis atclcc* mutant with *GhCLCc-1* rescued its salt-sensitive phenotype. In addition, the silencing of the *GhCLCc-1* gene led to an increased accumulation of Cl^−^ in the roots, stems, and leaves of cotton seedlings under salt treatments, resulting in compromised salt tolerance. And ectopic expression of the *GhCLCc-1* gene in *Arabidopsis* reduced the accumulation of Cl^−^ in transgenic lines under salt treatments, thereby enhancing salt tolerance. These findings elucidate that *GhCLCc-1* positively regulates salt tolerance by modulating Cl^−^ accumulation and could be a potential target gene for improving salt tolerance in plants.

## 1. Introduction

Salinization, in the past, was considered an environmental issue predominantly confined to arid regions [1,2,3]. However, over the last few decades, freshwater salinization has intensified in numerous global regions [4]. In contrast to primary or natural salinization, secondary salinization is a direct consequence of human activities. Among them, the use of chloride-based salt snow melting agents, such as sodium chloride (NaCl), potassium chloride (KCl), and calcium chloride (CaCl_2_), constitutes one of the significant factors contributing to the salinization of freshwater and arable land [5]. As one of the major abiotic stresses that limit crop growth and productivity, salt stress response genes have been widely identified and characterized in plants. The cytochrome P450 enzyme plays a role in the response of a variety of plants to salt stress [6,7]. In *Medicago truncatula*, transcription factor *MtHHO3* is involved in the ABA signaling pathway and negatively regulates salt tolerance [8]. The CmCIPK1-CmRbohD1/D2 complex enhances salt tolerance in pumpkin by modulating H_2_O_2_ signaling [9].

Ion toxicity induced by salt stress is primarily caused by the excessive accumulation of Na^+^ and Cl^−^ and the depletion of K^+^, leading to ionic imbalance [10,11]. For a long time, research on plant salinity stress has primarily focused on the toxic effects of cations. The salt tolerance mechanism, which encompasses the exclusion and compartmentalization of Na^+^ as well as K^+^ transport to maintain cellular Na^+^/K^+^ homeostasis, has been extensively and deeply investigated [12,13,14]. The mediation of Na^+^ efflux by the Na^+^/H^+^ antiporter Salt Overly Sensitive 1 (SOS1), which enhances plant salinity tolerance, has been confirmed in various plant species [15,16,17]. *STG5*, the key salt tolerance gene in rice, primarily participates in the regulation of the expression of multiple members of the HKT (High-affinity K^+^ Transporters) gene family, thereby controlling the Na^+^/K^+^ homeostasis and conferring enhanced salt tolerance to rice [18]. However, the salt damage induced by chloride ions (Cl^−^) has been largely neglected in many cases.

Cl^−^ is a micronutrient that is essential for all higher plants, primarily involved in stabilizing membrane potential, regulating intracellular pH gradients, and modulating electrical excitability [19,20]. Despite its beneficial roles in plant nutrition, the excessive accumulation of Cl^−^ within the plant can adversely affect plant growth and development [19]. The toxic effects of Cl^−^ on plants and the study of the chloride salt tolerance mechanisms have increasingly attracted attention. Some studies have demonstrated that controlling Cl^−^ transport from roots to shoots or maintaining low Cl^−^ in shoots is a critical factor in plant tolerance to chloride salt [21,22]. In maize, the cytokinin signaling pathway enhances the tolerance of salt by compartmentalizing Cl^−^ into the vacuoles of root cortex cells, thus reducing the transport of Cl^−^ to shoots [23].

Anion channels and transport proteins in plants play crucial roles in regulatory functions such as nutrient uptake, ion homeostasis, and resistance to biotic or abiotic stresses [24]. As an important Cl^−^ transporter, chloride channel proteins (CLCs) mediate Cl^−^ transport and homeostasis, which has garnered the attention of researchers [25]. In *Arabidopsis*, the CLC protein family consists of seven members, AtCLCa–g, which can be divided into two distinct classes: AtCLCa–d and AtCLCg belong to class I and AtCLCe and AtCLCf belong to class II [26]. Among them, AtCLCc and AtCLCg are considered to confer plant salt tolerance under chloride stress. Disruption of the *AtCLCc* gene seriously affects physiological processes linked to the movement of Cl^−^ across the tonoplast, disrupting Cl^−^ homeostasis and reducing salt tolerance [27]. *AtCLCg* has a high degree of identity with *AtCLCc*, but their functions are not redundant and they collectively form part of the regulatory network that controls chloride sensitivity [24]. In wild soybean, NaCl stress could induce heightened expression levels of *GsCLC-c2* in roots, enhancing salt tolerance through the sequestration of Cl^−^ accumulation in the root vacuoles [28]. *MhCLC-c1* mitigates cell death caused by NaCl through inhibiting intracellular Cl^−^ accumulation in *Malus hupehensis* [29]. Transgenic *Arabidopsis* can be tolerant to salt by overexpressing citrus *CsCLCc* [30]. Previously, we found that *GhCLCg-1* positively regulates salt tolerance in upland cotton by modulating Cl^−^ content and Na^+^/K^+^ ratio in roots, stems, and leaves [31].

This study identified and characterized *GhCLCc-1A/D*, the homologous genes of *Arabidopsis AtCLCc* in upland cotton. Through complementation in *Arabidopsis* mutant silencing in cotton and ectopic expression in *Arabidopsis*, we demonstrated that *GhCLCc-1* positively affects plant response to chloride salt stress.

## 2. Materials and Methods

### 2.1. Plant Materials and Treatments

Seeds of *Gossypium hirsutum* L. acc. TM-1 were planted in greenhouse pots containing vermiculite and covered with plastic film (16 h light/8 h dark, 23 °C). Seedlings showing uniform growth were selected when their cotyledons had fully expanded, and then transferred to hydroponic tanks equipped with aeration systems containing the Hoagland solution, with weekly replacement of the solution. The cotton seedlings with two true leaves were exposed to 150 mM NaCl or KCl Hoagland solution, with no NaCl or KCl added to the control (Mock). After treatment, roots, stems, and leaves were harvested at 0, 1, 3, 6, and 12 h, respectively. Further treatments were given to cotton seedlings with 0, 50, 100, 150, and 200 mM NaCl or KCl, respectively, and the roots were collected 6 h later.

*Arabidopsis* mutant and wild-type (WT) plants shared a Columbia (Col-0) background. The seeds of mutant *atclcc* (SALK_115644) were obtained from the NASC website (https://arabidopsis.info/BasicForm, accessed on 20 May 2023) and identified and consequently employed for phenotypic analysis [32]. The primer sequences for identifying mutants were designed using the online tool T-DNA Primer Design (http://signal.salk.edu/tdnaprimers.2.html, accessed on 25 May 2023) (Table S1). The seeds were positioned on Murashige and Skoog (MS) medium supplemented with 0 mM (Mock), 100 mM NaCl or KCl, and after 2 d of vernalization at 4 °C, they were transferred to the greenhouse (16 h light/8 h dark photoperiod, 21 °C). The length of the primary root was measured with a caliper, and the fresh weight and dry weight were also measured. Nine plants of each line were grouped together, with three biological replicates. Statistical analysis was performed in SPSS 21 using one-way analysis of variance (ANOVA) and Tukey’s HSD tests [33]. Following harvesting, the whole plants were analysed for Cl^−^, Na^+^, and K^+^ contents.

### 2.2. Cloning and Sequence Analyses

To clone the *CLCc* gene in upland cotton, *Arabidopsis* AtCLCc protein sequence was used as the query to search against the *Ghirsutum*_527_v2.1 genome database [34] using the BLAST program. As a result, *Gohir.A11G058300* (*GhCLCc-1A*) and *Gohir.D11G062600* (*GhCLCc-1D*) were identified as the *CLCc* genes in upland cotton. *GhCLCc-1* full-length coding sequence (CDS) was amplified with *GhCLCc-1*F/R primers designed by Oligo 7 (Table S1) [35].

A phylogenetic tree was built using the maximum likelihood (ML) method in MEGA7 software with the amino acid sequences of *Arabidopsis* and *G. hirsutum* (Table S2). Conserved domains were identified using the hmmscan search website (https://www.ebi.ac.uk/Tools/hmmer/search/hmmscan, accessed on 25 January 2024), and gene structures and domains were visualized using TBtools [36].

### 2.3. Quantitative Real-Time PCR (qRT-PCR)

Roots, stems, and leaves were subjected to total RNA extraction using the EASYspin Plus Complex Plant RNA Kit (Aidlab, Beijing, China). The HiScript II Q Select RT SuperMix for qPCR (+gDNA eraser) (Vazyme, Nanjing, China) was utilized to obtain first-strand cDNA. Gene-specific primers were tailored from the coding sequences of *GhCLCc-1A/D*. Subsequent qRT-PCR was carried out on the LightCycler 480 system (Roche, Basel, Switzerland) with ChamQ Universal SYBR qPCR Master Mix (Vazyme). The amplification program was as follows: 95 °C for 30 s; 40 cycles at 95 °C for 10 s, and 60 °C for 30 s; for the melting curve stage, the default settings were chosen. The 2^−∆CT^ method was used to calculate the relative expression of each target gene [37], with the *GhHis3* gene serving as the internal reference [38]. The primer sequences were designed by Oligo 7 and validated by electronic PCR (e-PCR) (Table S1) [35,39].

### 2.4. Subcellular Localization of GhCLCc-1A/D

The full-length CDS without the termination codon of *GhCLCc-1A* and *GhCLCc-1D* were amplified using primers with homologous arms (Table S1). These sequences were subsequently inserted into the *p*CAMBIA2300-GFP vector to generate green fluorescent proteins (GFP) GhCLCc-1A-GFP and GhCLCc-1D-GFP using the ClonExpress Ultra One Step Cloning Kit (Vazyme). The empty *p*CAMBIA2300-GFP vector served as a positive control, and λ-TIP-RFP served as the tonoplast marker with red fluorescent protein (RFP) [31]. The transient expression in *Arabidopsis* leaf mesophyll protoplasts was performed as previously described [40]. Following a dark incubation period of 12–20 h at room temperature, GFP and RFP signals were detected using a laser scanning confocal microscope (FV1200, Olympus, Tokyo, Japan).

### 2.5. Genetic Transformation

Due to the high sequence similarity between *GhCLCc-1A* and *GhCLCc-1D*, the coding region of *GhCLCc-1A* was amplified and cloned into the *p*CAMBIA2300 vector containing a constitutive promoter 35S. Then, the recombinant plasmid 35S::*GhCLCc-1A* was converted using the freeze–thaw transformation method into the *Agrobacterium tumefaciens* strain GV3101. A floral dip technique was used for transformation of recombinant plasmids into WT or mutant *atclcc* for overexpression and complementation experiments [41]. Semi-quantitative RT-PCR was performed on confirmed transgenic plants with *GhCLCc-1*-Q-F/R primers designed by Oligo 7, and *AtUBQ5* was used as a reference standard (Table S1) [42].

### 2.6. Virus-Induced Silencing (VIGS) of GhCLCc-1 in Upland Cotton

A 300 bp fragment was amplified with *GhCLCc-1*-p-F/R primers based on the conserved sequences of *GhCLCc-1A* and *GhCLCc-1D* (Table S1) and inserted into the pTRV2 vector using the ClonExpress Ultra One Step Cloning Kit (Vazyme). *Agrobacterium* cultures carrying pTRV1 were mixed with equivalent amounts of TRV:00, TRV:*CLA*, and TRV:*GhCLCc-1*, respectively. A syringe was used to introduce each mixture into the cotyledons when the cotyledons were fully expanded. When the cotton seedlings exhibited an albino phenotype, the silencing efficiency of *GhCLCc-1* was assessed by qRT-PCR. Chloride salt treatments were applied to cotton seedlings at the two-leaf stage, and the roots, stems, and leaves were collected to analyze the content of Cl^−^, Na^+^, and K^+^, respectively.

### 2.7. Biochemical Index Measurement

The levels of hydrogen peroxide (H_2_O_2_), malondialdehyde (MDA), and chlorophyll in cotton seedling leaves were determined using the Hydrogen Peroxide Content Assay Kit (Solarbio, Beijing, China), Malondialdehyde (MDA) Content Assay Kit (Solarbio), and Chlorophyll Assay Kit (Solarbio) according to the manufacturer’s instructions, respectively. All the analyses were carried out in triplicate.

### 2.8. Analysis of Ion Contents

In order to denature enzymes, cotton and *Arabidopsis* samples were collected and incubated at 105 °C for 10 min, followed by incubation at 75 °C until a constant weight was achieved. A powder was then created by grinding the samples. For Cl^−^ content determination, a 50 mL Erlenmeyer flask was initially rinsed with 5% dilute nitric acid (HNO_3_) and subsequently dried. Then, 0.1 g of sample powder and 15 mL of deionized water were added to the flask, which was boiled for 10 min before cooling to room temperature. Filter paper was used to filter the solution into a 25 mL volumetric flask, and the residue was rinsed thrice with deionized water. The volume was finally adjusted to 25 mL with deionized water. After filtering the liquid through a 0.45 μm microporous membrane filter, the Cl^−^ content was examined via an Ion Chromatography (IC) system (ICS-5000, Thermo Fisher Scientific, Waltham, MA, USA) [31].

For Na^+^ and K^+^ content determination, the dried sample powder was sieved through a 100-mesh nylon screen. Approximately 0.1 g of the sieved plant powder was thoroughly digested on a microwave digester using HNO_3_, hydrofluoric acid (HF), and H_2_O_2_. Following acid digestion, the acids in the sample were expelled using an acid evaporator, and the sample was brought to volume with deionized water. The contents of Na^+^ and K^+^ were then analyzed in the solution after filtering through a 0.45 μm microporous membrane filter using an Inductively Coupled Plasma Optical Emission Spectrometry (ICP-OES) system (ICAP7400, Thermo Fisher Scientific, USA) [43]. Each sample was analyzed in three biological replicates.

## 3. Results

### 3.1. GhCLCc-1 Transcript Abundance Is Induced by Chloride Salt Stress

Two homologous genes, *GhCLCc-1A* and *GhCLCc-1D*, were identified and cloned in upland cotton using *AtCLCc* gene as the query sequence. CLCs can be divided into two clades based on evolutionary analysis, with GhCLCc-1 belonging to Class I, exhibiting high homology with AtCLCc (Figure S1A). In addition, structural analysis revealed that *GhCLCc-1A* and *GhCLCc-1D* have highly similar gene structures, with an identical arrangement and quantity of introns and exons, consisting of seven exons and six introns each. Furthermore, both genes contained the voltage_CLC and CBS domains specific to the CLC family (Figure S1B).

**Figure 1:**
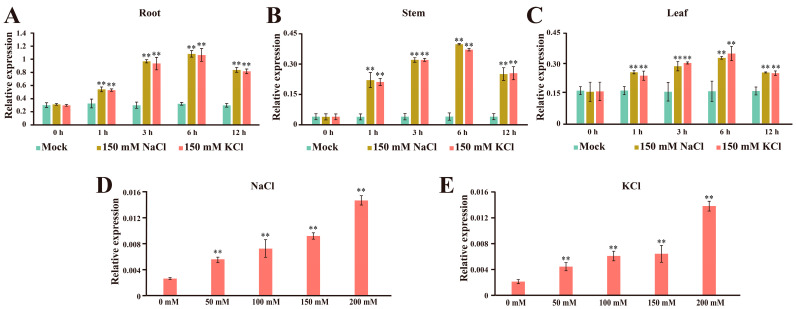
Expression patterns of *GhCLCc-1* in cotton seedlings under salt treatment. At 0, 1, 3, 6, and 12 h after being treated with 0 mM (Mock), 150 mM NaCl, or 150 mM KCl, the expression levels were evaluated in (**A**) roots, (**B**) stems, and (**C**) leaves. The expression levels were evaluated in roots at 6 h after being treated with 0, 50, 100, 150, and 200 mM (**D**) NaCl or (**E**) KCl. Error bars indicate the standard deviation (SD) of three biological replicates (Student’s *t*-test; ** *p* < 0.01).

To investigate the transcription levels of the *GhCLCc-1A/D* gene under chloride salt treatment, the expression levels of the *GhCLCc-1A/D* gene were detected using a pair of shared primers via qRT-PCR after treatment with 150 mM NaCl or KCl. The results showed that treatments with chloride salt significantly induced the expression of *GhCLCc-1*, especially in the roots, and peaked at 6 h post-treatment (Figure 1A–C). Subsequently, cotton seedlings were treated with chloride at different concentrations, and the expression level of *GhCLCc-1* gradually increased as NaCl concentration increased (Figure 1D). A similar trend was also observed under KCl treatment (Figure 1E). These results indicate that the *GhCLCc-1* gene is highly conserved during evolution and may be responsible for responding to chloride salt stress.

### 3.2. GhCLCc-1 Localizes to the Tonoplast

**Figure 2:**
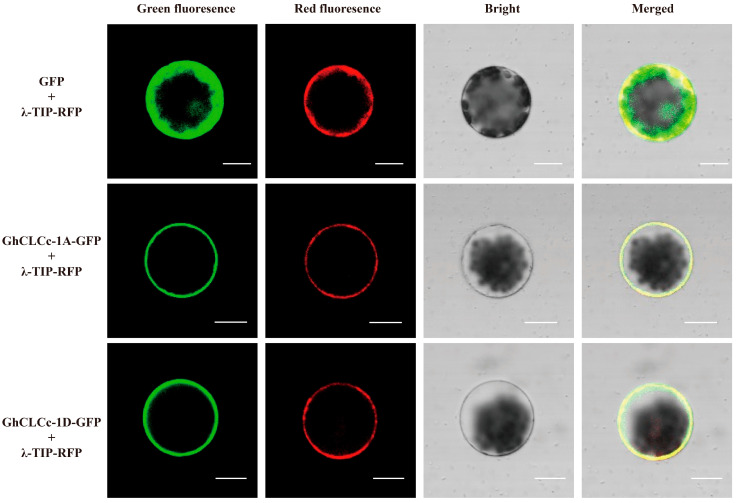
Subcellular localization of GhCLCc-1 in *Arabidopsis* leaf protoplasts. GhCLCc-1A-GFP and GhCLCc-1D-GFP were respectively co-expressed with the tonoplast marker protein λ-TIP-RFP. GFP co-transformed with λ-TIP-RFP served as the positive control. Scale bars = 10 μM.

Subcellular localization of the GhCLCc-1 protein was performed with the GhCLCc-1A-GFP and GhCLCc-1D-GFP vectors. The fusion proteins and GFP control protein were separately introduced into *Arabidopsis* mesophyll protoplasts together with the tonoplast marker protein λ-TIP-RFP and then transiently expressed. Confocal microscopy examination showed that the GhCLCc-1A/D-GFP signals overlapped with the λ-TIP-RFP signal. The control group exhibited widespread distribution of green fluorescence (Figure 2). It is evident from these results that GhCLCc-1A and GhCLCc-1D have tonoplast localizations.

### 3.3. GhCLCc-1 Rescues Chlorine Salt Tolerance in Arabidopsis Mutant Atclcc

**Figure 3:**
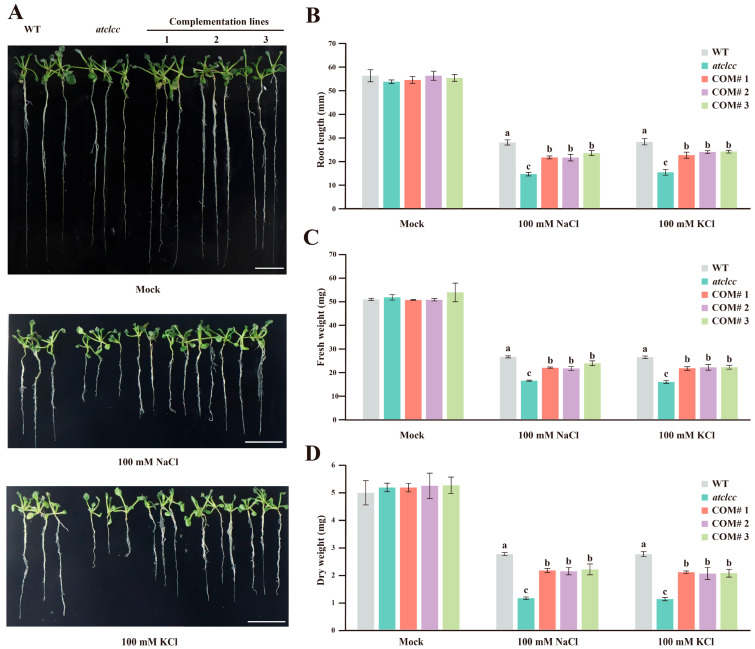
Phenotype and physiological parameters of wild type, *atclcc-1* mutant, and complementation lines. (**A**) Phenotype, (**B**) root length, (**C**) fresh weight, and (**D**) dry weight of *Arabidopsis* after treatment with 0 mM (Mock), 100 mM NaCl, or 100 mM KCl for 14 d. Scale bar = 10 mm. Error bars indicate the standard deviation (SD) of three biological replicates. Statistical analysis was performed using one-way ANOVA and Tukey’s HSD test. Different letters indicate significant differences (*p* < 0.05).

To investigate the function of *GhCLCc-1*, a vector containing *GhCLCc-1* was introduced into the *atclcc* mutant to generate complementary lines (COM) (Figure S2). The growth status of WT, *atclcc* mutants, and complemented lines was not significantly different after 14 d of germination on MS medium (Figure 3A). However, when cultured on medium containing 100 mM NaCl or KCl, WT and complemented lines exhibited longer roots and enhanced salt tolerance compared with the *atclcc* mutants, and the chlorine salt-sensitive phenotype of mutant lines was rescued in the complementary lines (Figure 3A,B). The fresh and dry weights of treated *Arabidopsis* plants were entirely consistent with the root length (Figure 3C,D). These results indicate that ectopic expression of *GhCLCc-1* partially restores tolerance to chloride salt stress in *Arabidopsis* mutants.

### 3.4. Silencing GhCLCc-1 in Cotton Reduces Chlorine Salt Tolerance

To further explore the role of *GhCLCc-1* in cotton, *GhCLCc-1*-silenced plants were generated using the VIGS system. Ten days after *Agrobacterium* infiltration, positive control TRV:*CLA* plants exhibited an albino phenotype (Figure S3A). Afterwards, a distinctly lower expression of *GhCLCc-1* in the TRV:*GhCLCc-1* plants was monitored (Figure S3B), indicating effective silencing of *GhCLCc-1*.

**Figure 4:**
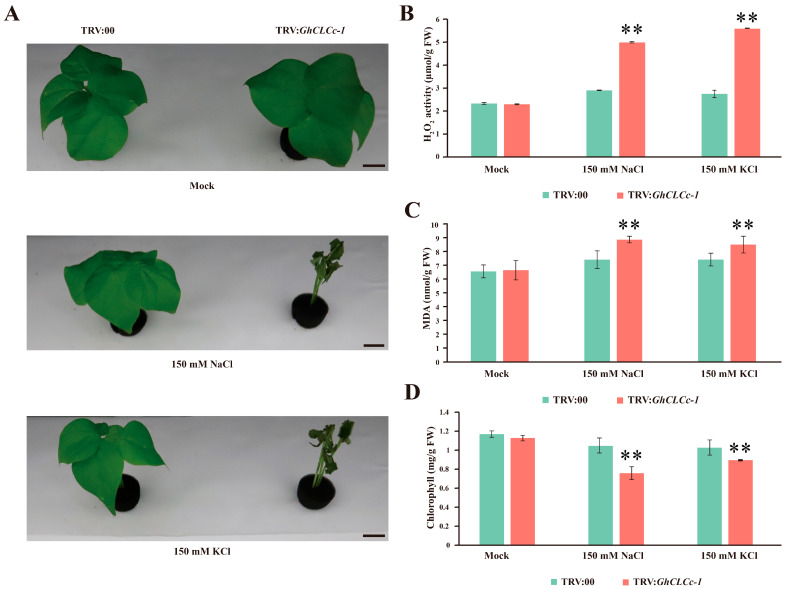
Effect of silencing *GhCLCc-1* on cotton seedlings. (**A**) Phenotype, (**B**) H_2_O_2_ content, (**C**) MDA content, and (**D**) chlorophyll content of TRV:00 and TRV:*GhCLCc-1* plants after treatment with 0 mM (Mock), 150 mM NaCl, or 150 mM KCl for 4 d. Scale bar = 2 cm. Error bars indicate the standard deviation (SD) of three biological replicates (Student’s *t*-test; ** *p* < 0.01).

**Figure 5:**
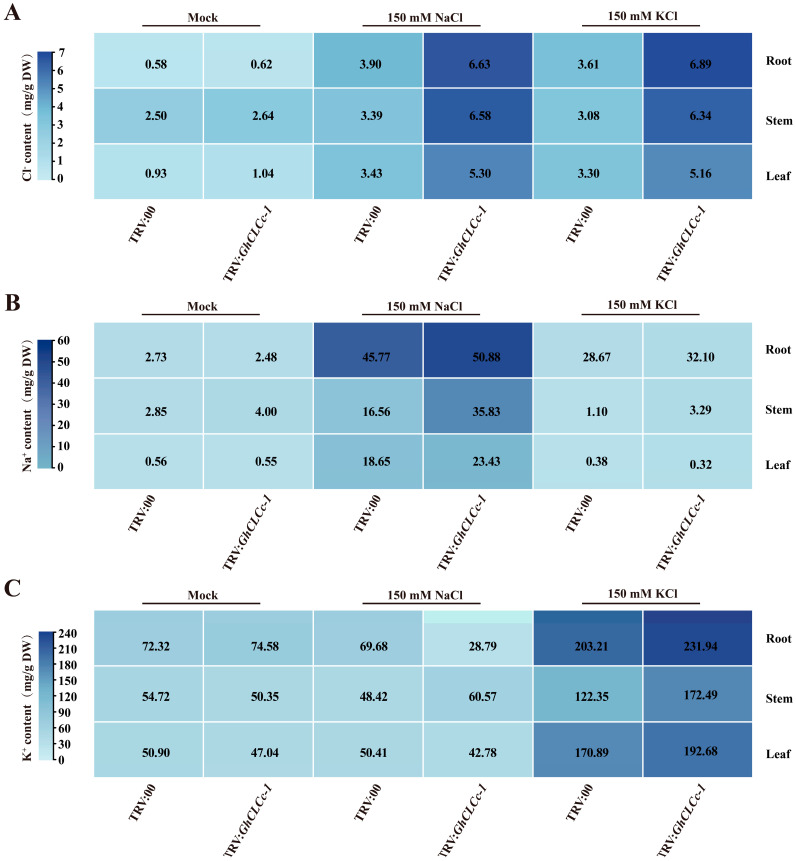
Ion content of TRV:00 and TRV:*GhCLCc-1* plants. (**A**) Cl^−^ content, (**B**) Na^+^ content, and (**C**) K^+^ content of roots, stems, and leaves after treatment with 0 mM (Mock), 150 mM NaCl, or 150 mM KCl for 4 d.

Under normal growth conditions, there were no discernible disparities between TRV:00 and TRV:*GhCLCc-1* plants. However, TRV:*GhCLCc-1* plants displayed an intensified salt-sensitive phenotype, predominantly characterized by leaf wilting and abscission after a 4 d treatment with 150 mM NaCl or KCl, in contrast to TRV:00 plants (Figure 4A). Under environmental stress, plants often undergo physiological and biochemical variation to adapt to environment. Therefore, we further assessed the levels of H_2_O_2_, MDA, and chlorophyll in the leaves TRV:00 and TRV:*GhCLCc-1* plants under two different chloride salt stresses. The levels of H_2_O_2_ and MDA were significantly increased in TRV:*GhCLCc-1* plants after NaCl and KCl treatments (Figure 4B,C), while the chlorophyll content was significantly reduced (Figure 4D). These findings indicated that chloride salt stresses lead to an increased production of reactive oxygen species (ROS), causing oxidative stress, which damages the cellular membrane and impairs photosynthetic capacity. The measurement results of Cl^−^, Na^+^, and K^+^ contents in the roots, stems, and leaves of cotton seedlings showed that, compared to TRV:00, the Cl^−^ content in TRV:*GhCLCc-1* plants was significantly higher after chlorine salt treatments (Figure 5A). Concurrently, the levels of the respective cations significantly increased in TRV:*GhCLCc-1* plants following NaCl or KCl treatment, indicating that TRV:*GhCLCc-1* plants were subjected to ionic stress induced by the salt treatments (Figure 5B,C). These results indicate that the silencing of *GhCLCc-1* exacerbates oxidative stress and ion accumulation in plants under chloride salt treatment, ultimately leading to a diminished salt stress tolerance in cotton.

### 3.5. GhCLCc-1 Ectopic Expression Enhances Chloride Salt Tolerance in Arabidopsis

**Figure 6:**
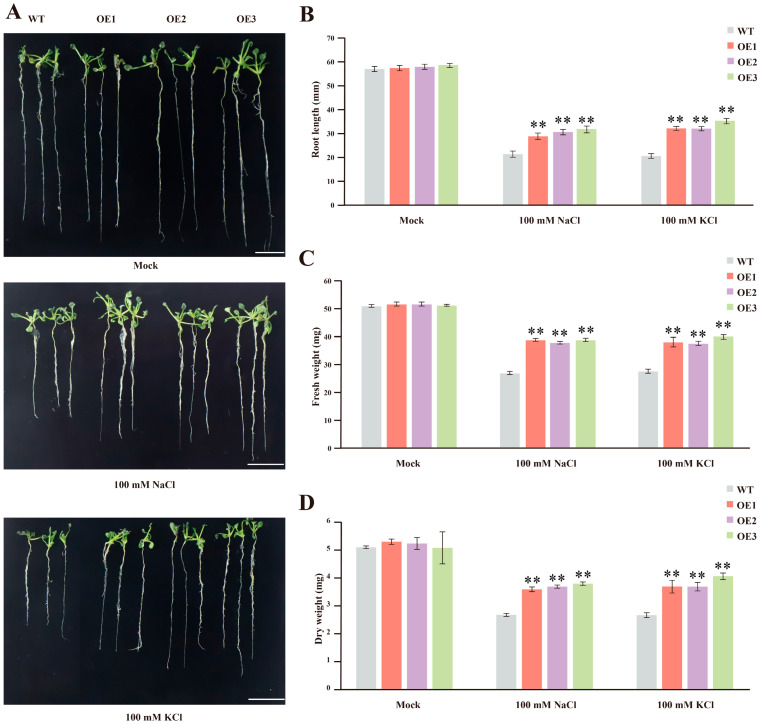
Effect of ectopic expression of *GhCLCc-1* on *Arabidopsis*. (**A**) Phenotype, (**B**) root length, (**C**) fresh weight, and (**D**) dry weight of *Arabidopsis* after treatment with 0 mM (Mock), 100 mM NaCl, or 100 mM KCl for 14 d. Scale bar = 10 mm. Error bars indicate the standard deviation (SD) of three biological replicates (Student’s *t*-test; ** *p* < 0.01).

**Figure 7:**
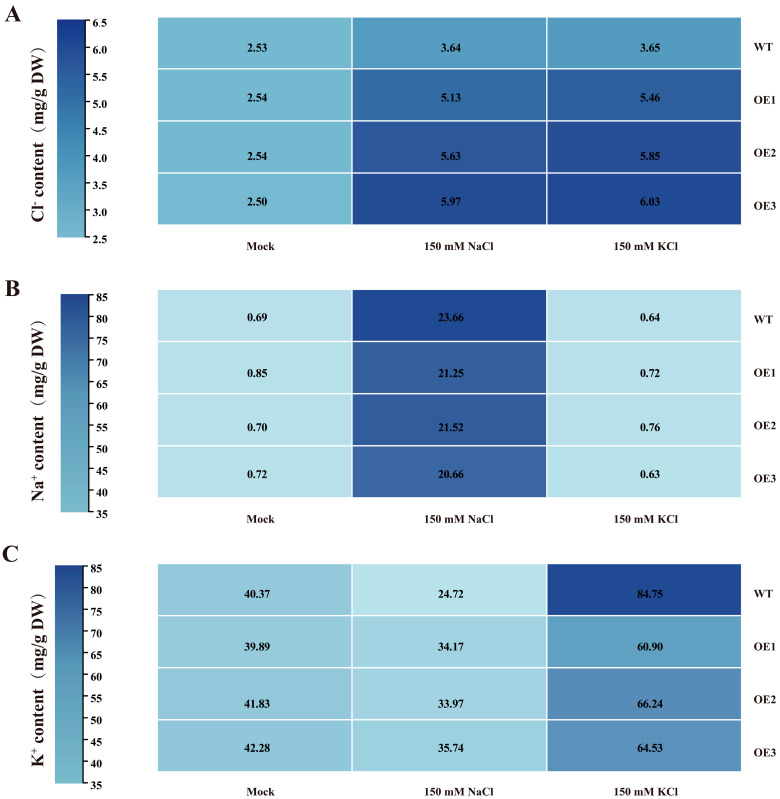
Ion content of WT and transgenic *Arabidopsis*. (**A**) Cl^−^ content, (**B**) Na^+^ content, and (**C**) K^+^ content of *Arabidopsis* seedlings after treatment with 0 mM (Mock), 100 mM NaCl, or 100 mM KCl for 14 d.

The function of the *GhCLCc-1* gene was further confirmed through ectopic expression in *Arabidopsis* (Figure S4). All plants exhibited similar phenotypes on MS medium. However, *GhCLCc-1* overexpressed *Arabidopsis* exhibited enhanced salt tolerance, as evidenced by larger leaves and longer roots compared to WT plants after 14 d of treatment with 100 mM NaCl or KCl (Figure 6A,B). Fresh weight and dry weight measurements indicated that *GhCLCc-1* overexpressed *Arabidopsis* lines had higher fresh weights and dryer weights than WT lines (Figure 6C,D). The determination of Cl^−^, Na^+^, and K^+^ contents in the *Arabidopsis* seedlings revealed that, compared to WT, the Cl^−^ content significantly decreased in transgenic *Arabidopsis* lines after treatment with 100 mM NaCl or KCl (Figure 7A). Additionally, following NaCl or KCl treatment, the levels of respective cations significantly increased in all the tested plants, but slightly decreased in transgenic lines compared with WT (Figure 7B,C). These findings suggest that *GhCLCc-1* ectopic expression improves *Arabidopsis* resistance to chloride stress by modulating ion levels.

## 4. Discussion

Plant growth and development are negatively affected by ionic toxicity induced by salt stress, leading to reduced crop yields. Chloride ions (Cl^−^), due to their limited ability to form complexes and their dependence on water flow for movement within the soil, are readily absorbed by plant roots, thereby exacerbating ionic toxicity [44]. Here, we identified and characterized two *AtCLCc* homologs (*GhCLCc-1A* and *GhCLCc-1D*) from upland cotton, and revealed that *GhCLCc-1* positively regulates plant tolerance to chlorides. Phylogenetic analysis revealed that *GhCLCc-1A/D*, along with *AtCLCc* and *AtCLCg*, are situated on the same evolutionary branch and contain the typical CLC family structural domains voltage_CLC and CBS [45,46], suggesting they may possess functions similar to those of *AtCLCc* and *AtCLCg*. The transcript abundance of *GhCLCc-1* was rapidly induced by NaCl or KCl treatments, with a significant increase in expression observed as early as 1 h post-treatment, peaking at 6 h (Figure 1). Furthermore, the expression levels of *GhCLCc-1A/D* gradually increased with the rising concentrations of NaCl or KCl treatments. The expression pattern analyses indicate that *GhCLCc-1* is involved in the response of chloride treatment in cotton.

Maintenance of Cl^−^ homeostasis is an important mechanism of chloride tolerance in plants [47]. In higher plants, the tonoplast is permeable to Cl^−^, utilizing ion transport proteins on the tonoplast to compartmentalize Cl^−^ into the vacuole, thereby reducing the cytosolic Cl^−^ concentration, thereby mitigating the toxic effects of Cl^−^ on the cell [48,49]. Similar to AtCLCc [27], GhCLCc-1A and GhCLCc-1D were both localized to the tonoplast of *Arabidopsis* protoplasts (Figure 2), implying their potential roles in transporting excess Cl^−^ from the cytoplasm into the vacuole. To investigate the possible role of *GhCLCc-1* in chloride salt tolerance, we generated complementation lines by ectopically expressing *GhCLCc-1* in *Arabidopsis atclcc* mutant, which is sensitive to salt stress. Compared to the *atclcc* mutant, the complementation lines exhibited significant increases in root length, dry weight, fresh weight, although they were not as high as in wild species, the strains showed salt tolerance, the strains showed salt tolerance (Figure 3). The findings indicate that *GhCLCc-1* is functionally analogous to *AtCLCc* and can rescue the phenotype of the mutant. This is consistent with the finding that ectopic expression of soybean CLCs enhances NaCl tolerance in BY2 cells and *Arabidopsis* [50,51].

Similar to *Arabidopsis atclcc* mutants, silencing *GhCLCc-1* in cotton also resulted in salt-sensitive phenotypes in seedlings (Figure 4). Under chloride salt stresses, higher levels of H_2_O_2_ and MDA, along with reduced chlorophyll content, were detected in TRV:*GhCLCc-1* plants. H_2_O_2_ is a particularly stable ROS. Salt stress-induced excessive accumulation of ROS can damage the cell membrane, leading to intensified lipid peroxidation of the cell membrane and increased production of MDA, ultimately resulting in plant cell damage [52,53]. Chlorophyll content is one of the few physiological parameters closely associated with salt tolerance, and salt stress accelerates the degradation of chlorophyll in plants, leading to a reduction in photosynthesis [54,55]. A study on the halophyte *Suaeda altissima* implies that the increased expression of *SaCLCd*, *SaCLCf*, and *SaCLCg* were associated with Cl^−^ accumulation in leaf cells [56]. Here, the silencing of *GhCLCc-1* resulted in the increased accumulation of Cl^−^ and cations in the roots, stems, and leaves of cotton under chloride salt treatment, thereby reducing the salt tolerance of cotton (Figure 5). According to these results, *GhCLCc-1* is critical for the uptake and transport of Cl^−^ in cotton. Furthermore, *GhCLCc-1* ectopic expression significantly enhanced transgenic *Arabidopsis* chloride salt tolerance (Figure 6), indicating the potential applicability of *GhCLCc-1* in other crops. Ectopic expression of trifoliate orange *CsCLCc* in *Arabidopsis* reduced Cl^−^ accumulation in the roots and shoots of transgenic plants under NaCl treatment [30]. In our study, consistently, transgenic plants exhibited significantly lower accumulation of Cl^−^ compared to the WT under chloride salt stresses. Additionally, there was a decrease in Na^+^ and K^+^ levels as well (Figure 7). At the tissue level, anion efflux channels release Cl^−^ from plant cells to limit the net Cl^−^ uptake, thus inhibiting the ion’s entry into the plant and its subsequent transportation to the shoots [44,57]. Therefore, we hypothesize that *GhCLCc-1* may be involved in the efflux of Cl^−^ to mitigate the toxic effects of Cl^−^, thereby enhancing the chloride salt tolerance of transgenic *Arabidopsis*. These results indicate that the *GhCLCc-1* plays a significant role in the homeostasis of Cl^−^ and is a crucial genetic target for enhancing plant salt tolerance.

## 5. Conclusions

In summary, we cloned and characterized two CLC genes *GhCLCc-1A/D* from upland cotton. GhCLCc-1A/D were localized to the tonoplast, and their transcription were significantly induced by chloride salts. Subsequently, by complementing the *GhCLCc-1* gene in *Arabidopsis atclcc* mutants, silencing the *GhCLCc-1* gene in cotton, and ectopically expressing the *GhCLCc-1* gene in *Arabidopsis*, it was demonstrated that the *GhCLCc-1* gene positively regulates plant salt stress tolerance through modulating the accumulation of Cl^−^ within the plant. Our data clearly indicate that the upland cotton *GhCLCc-1* gene plays an important role in the plant’s response to salinity by mitigating the toxic effects of Cl^−^.

## Data Availability

The original contributions presented in the study are included in the article and Supplementary Materials, further inquiries can be directed to the corresponding author.

## Supplementary Materials

The following supporting information can be downloaded at: https://www.mdpi.com/article/10.3390/genes15050555/s1, Figure S1: Phylogenetic analysis and structure feature of GhCLCc-1A/D. (A) Phylogenetic tree of GhCLCc-1A/D and AtCLCs. (B) Intron-exon structure and conserved domains of GhCLCc-1A/D; Figure S2: Identification of Arabidopsis atclcc mutants and complementation lines. (A) Identification of atclcc mutants. (B) Identification of complementation lines; Figure S3: Silencing efficiency of GhCLCc-1 in cotton seedlings. (A) The albino phenotype of positive control TRV:CLA plants. (B) Relative expression of GhCLCc-1 in TRV:00 and TRV:GhCLCc-1 plants. Scale bar = 2 cm, Student’s t-test; ** *p* < 0.01; Figure S4. Identification of transgenic Arabidopsis; Table S1: Primers used in this study; Table S2: Genes used in this study.
