# Supplementary figures and images for: GhCLCc-1, a Chloride Channel Gene from Upland Cotton, Positively Regulates Salt Tolerance by Modulating the Accumulation of Chloride Ions
Source: Genes (Basel). 2024 Apr 26;15(5):555. doi: 10.3390/genes15050555 (PMC11120929; doi:10.3390/genes15050555)

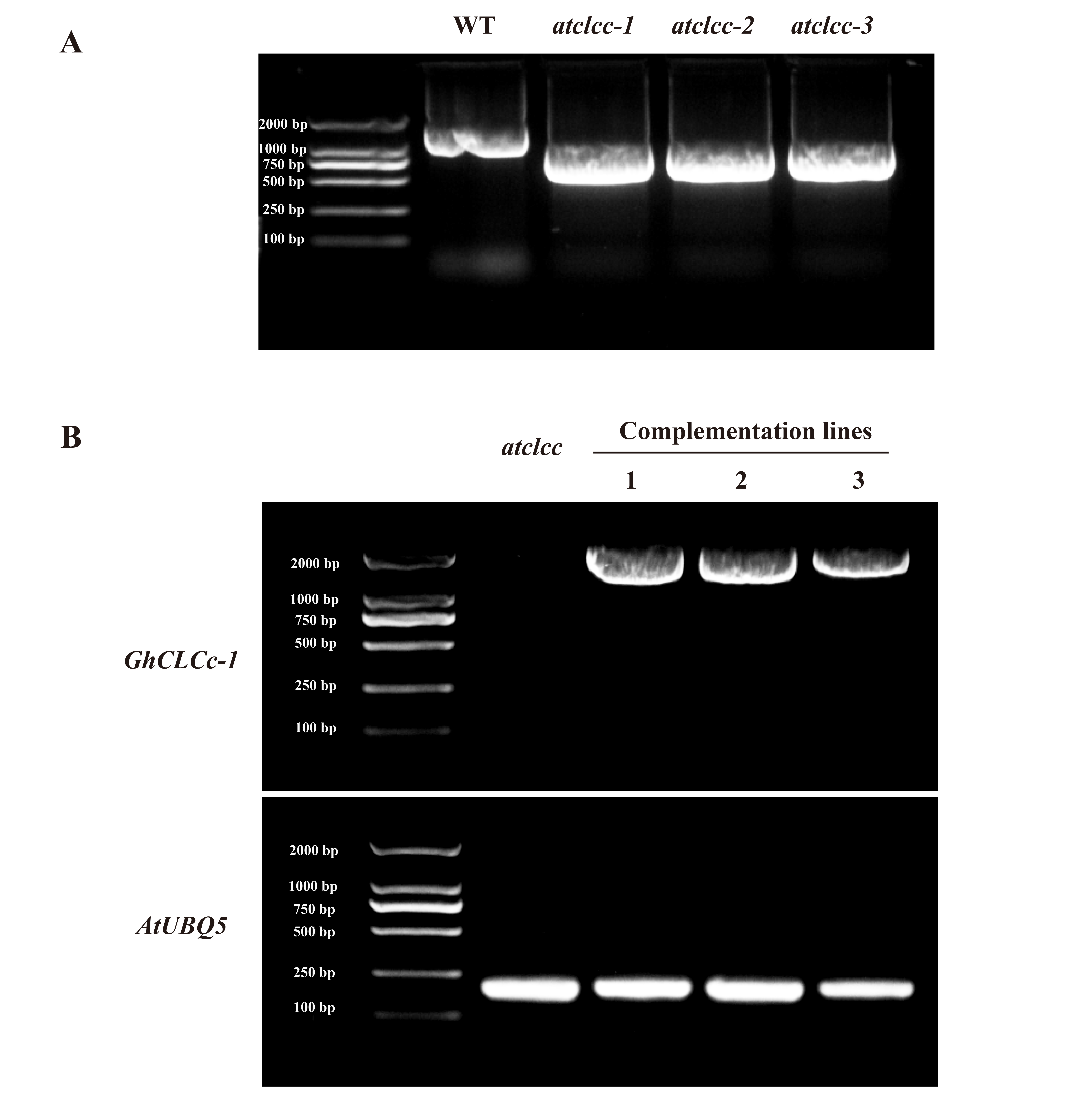

Supplement: Supplementary file 1 [file genes-15-00555-s001.zip › Figure S2.tif]

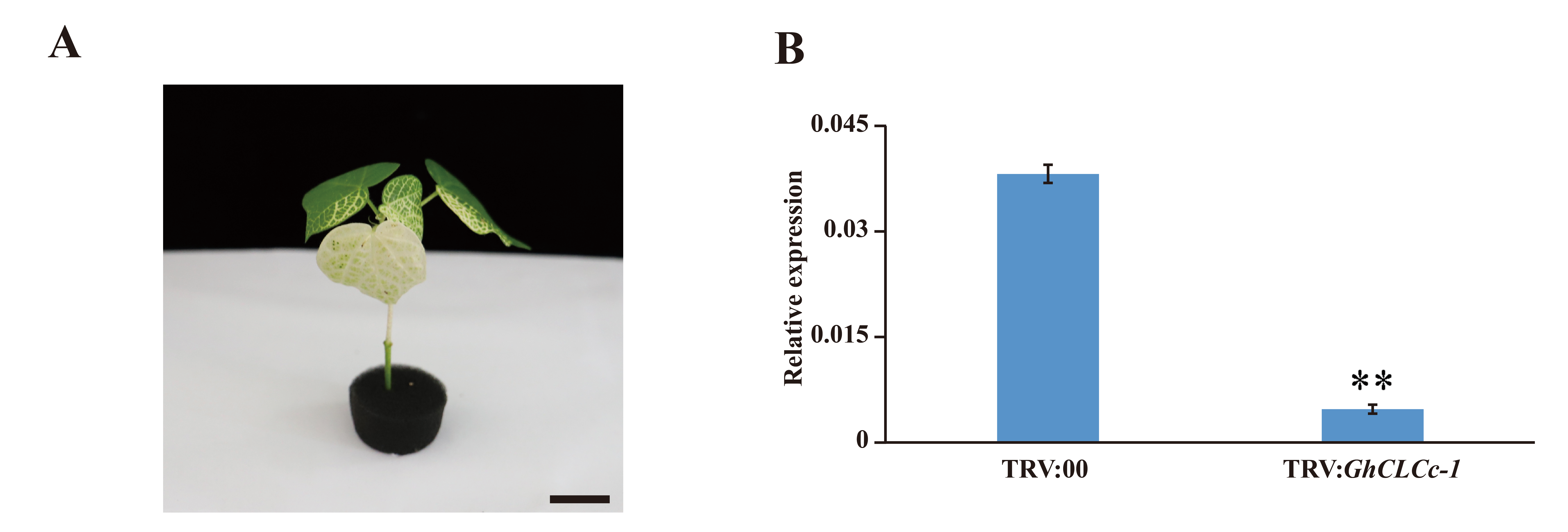

Supplement: Supplementary file 1 [file genes-15-00555-s001.zip › Figure S3.tif]

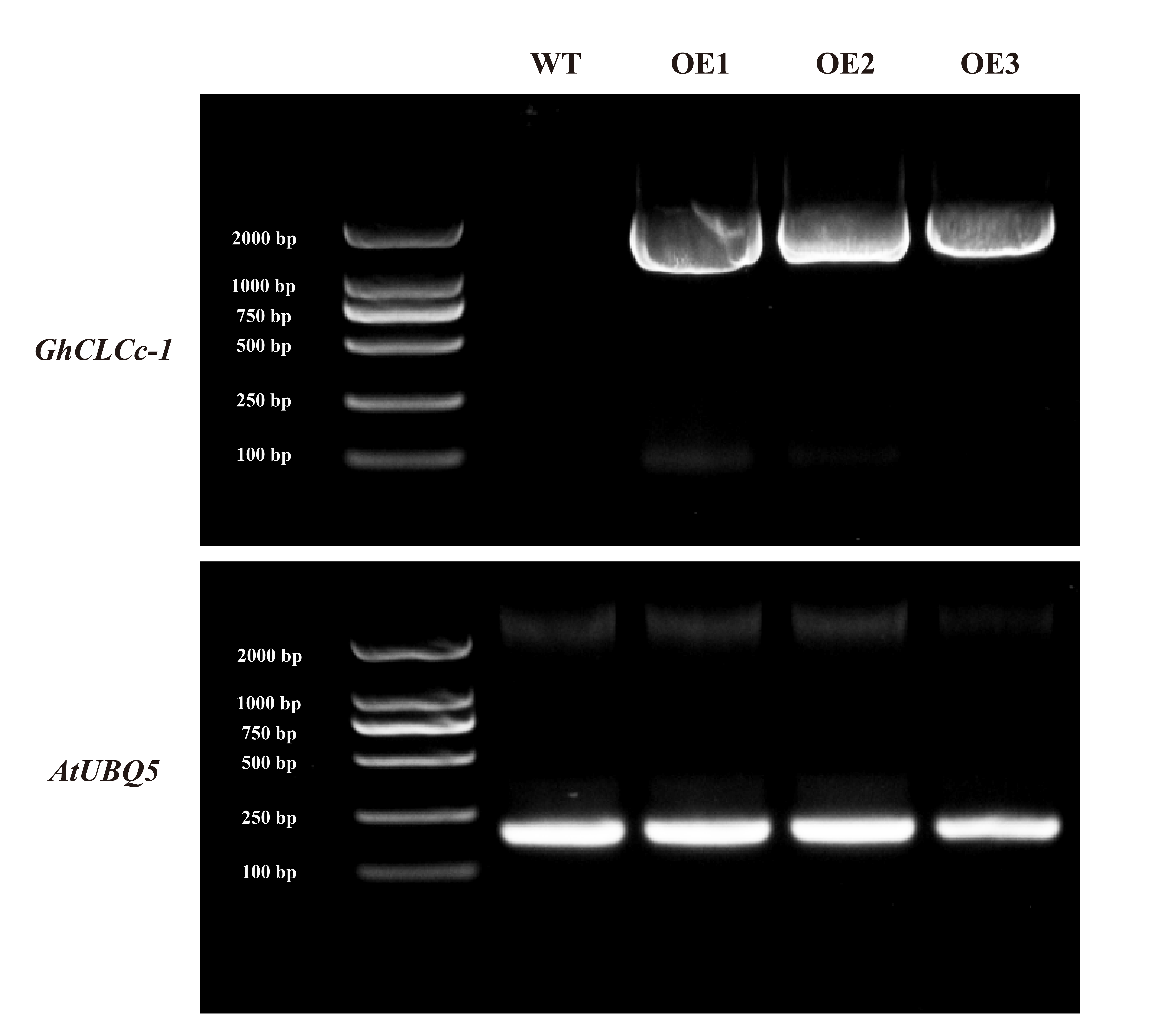

Supplement: Supplementary file 1 [file genes-15-00555-s001.zip › Figure S4.tif]

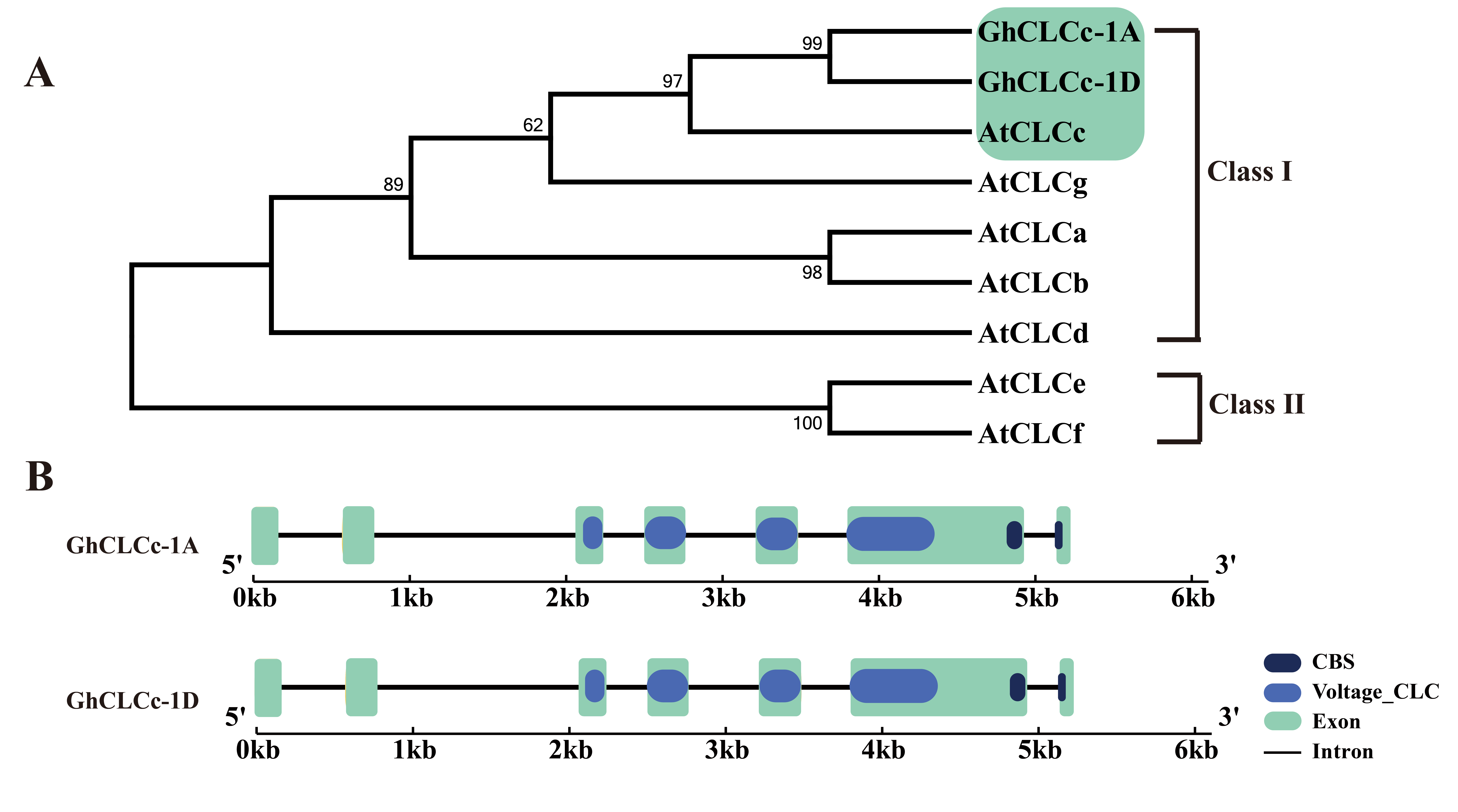

Supplement: Supplementary file 1 [file genes-15-00555-s001.zip › Figure S1.tif]
